# Small airway hyperresponsiveness in COPD: relationship between structure and function in lung slices

**DOI:** 10.1152/ajplung.00325.2018

**Published:** 2019-01-10

**Authors:** Harm Maarsingh, Cécile M. Bidan, Bindi S. Brook, Annet B. Zuidhof, Carolina R.S. Elzinga, Marieke Smit, Anouk Oldenburger, Reinoud Gosens, Wim Timens, Herman Meurs

**Affiliations:** ^1^Department of Molecular Pharmacology, University of Groningen, Groningen, The Netherlands; ^2^Department of Pharmaceutical Sciences, Lloyd L. Gregory School of Pharmacy, Palm Beach Atlantic University, West Palm Beach, Florida; ^3^Laboratoire Interdisciplinaire de Physique, Centre for Scientific Research, Université Grenoble Alpes, Grenoble, France; ^4^Department of Biomaterials, Max Planck Institute of Colloids and Interfaces, Potsdam, Germany; ^5^School of Mathematical Sciences, University of Nottingham, Nottingham, United Kingdom; ^6^Department of Pathology and Medical Biology, University Medical Center Groningen, Groningen, The Netherlands; ^7^Groningen Research Institute of Asthma and Chronic Obstructive Pulmonary Disease, University Medical Center Groningen, University of Groningen, Groningen, The Netherlands; ^8^Groningen Research Institute of Pharmacy, University of Groningen, Groningen, The Netherlands

**Keywords:** airway constriction, airway remodeling, biomechanical modeling, emphysema, human lung

## Abstract

The direct relationship between pulmonary structural changes and airway hyperresponsiveness (AHR) in chronic obstructive pulmonary disease (COPD) is unclear. We investigated AHR in relation to airway and parenchymal structural changes in a guinea pig model of COPD and in COPD patients. Precision-cut lung slices (PCLS) were prepared from guinea pigs challenged with lipopolysaccharide or saline two times weekly for 12 wk. Peripheral PCLS were obtained from patients with mild to moderate COPD and non-COPD controls. AHR to methacholine was measured in large and small airways using video-assisted microscopy. Airway smooth muscle mass and alveolar airspace size were determined in the same slices. A mathematical model was used to identify potential changes in biomechanical properties underlying AHR. In guinea pigs, lipopolysaccharide increased the sensitivity of large (>150 μm) airways toward methacholine by 4.4-fold and the maximal constriction of small airways (<150 μm) by 1.5-fold. Similarly increased small airway responsiveness was found in COPD patients. In both lipopolysaccharide-challenged guinea pigs and patients, airway smooth muscle mass was unaltered, whereas increased alveolar airspace correlated with small airway hyperresponsiveness in guinea pigs. Fitting the parameters of the model indicated that COPD weakens matrix mechanical properties and enhances stiffness differences between the airway and the parenchyma, in both species. In conclusion, this study demonstrates small airway hyperresponsiveness in PCLS from COPD patients. These changes may be related to reduced parenchymal retraction forces and biomechanical changes in the airway wall. PCLS from lipopolysaccharide-exposed guinea pigs may be useful to study mechanisms of small airway hyperresponsiveness in COPD.

## INTRODUCTION

Chronic obstructive pulmonary disease (COPD) is a chronic inflammatory disease, characterized by a progressive and partially irreversible decline in lung function and by airway hyperresponsiveness (AHR) (13, 14, 23, 24). Chronic inflammation in COPD causes structural alterations and narrowing of particularly the small airways, and emphysema, characterized by parenchymal destruction. It has been proposed that loss of lung function and AHR may result from small airway remodeling, including increased airway smooth muscle (ASM) mass, and from loss of elastic recoil by parenchymal damage (13, 14, 23, 24). These structural changes may differ between patients, depending on various factors (13).

Although this hypothesis has been around for several decades, evidence for a direct relationship between structural changes in the lung and small airway function is still lacking. Measurement of airway mechanics in precision-cut lung slices (PCLS) using video-assisted microscopy ex vivo (19, 26) may be highly instrumental in investigating the impact of structural changes on airway responsiveness. Microscopic visualization allows performance of these studies even in the smallest airways, a major breakthrough for the study of small airway function.

Limited studies on small airway mechanics in PCLS from animal models of COPD are known (5, 7, 8, 16, 17, 33). In one of these studies, enhanced small airway responsiveness to carbachol and serotonin was found in rats after chronic in vivo exposure to tobacco smoke, which was associated with increased airway wall α-smooth muscle actin (α-SMA) content (5). In contrast, no small airway hyperresponsiveness was observed in mice after short-term exposure to cigarette smoke (8), elastase (17), or lipopolysaccharide (LPS) (7). However, exposure of mouse PCLS to elastase or collagenase ex vivo induced AHR to acetylcholine and methacholine (16, 17, 33). Mechanical studies in PCLS from patients with COPD have thus far not been described.

In this study, we investigated alterations in the responsiveness of large and small intrapulmonary airways to methacholine in PCLS obtained from a guinea pig model of COPD induced by chronic LPS exposure (22). This model resembles COPD patho(physio)logy in several ways: the presence of neutrophilic inflammation, mucus hypersecretion, emphysema, small airway fibrosis, and vascular remodeling (22). In addition, guinea pig lungs contain both large and small airways. We also determined the relationship between structural components of the airways and parenchyma (ASM mass and parenchymal integrity) and responsiveness of the individual airways within the same PCLS. Importantly, this relationship was also assessed for small airways in lung slices obtained from human control subjects and patients with mild to moderate COPD. Finally, we used a multiscale model of an airway embedded in parenchyma (12) to identify possible alterations in biomechanical properties that could underlie the observations in PCLS.

## METHODS

### 

#### Animals.

Outbred male specified pathogen-free Dunkin Hartley guinea pigs (Harlan, Heathfield, UK) weighing 350–400 g were used. The animals were housed in pairs under a 12-h:12-h light-dark cycle in a temperature- and humidity-controlled room with food and tap water ad libitum. All animal care and experimental procedures complied with the animal protection and welfare guidelines, were approved by the Institutional Animal Care and Use Committee of the University of Groningen, The Netherlands, and are reported in compliance with the ARRIVE guidelines (18).

#### Lipopolysaccharide instillation.

At the start of the protocol, guinea pigs were randomly selected to be challenged by intranasal instillation of 200 μl LPS (5 mg/ml in sterile saline; Sigma-Aldrich, St. Louis, MO) or 200 μl sterile saline (control group) two times weekly for 12 consecutive weeks as described by Pera et al. (22). To this aim, conscious guinea pigs were held in an upright position while the LPS solution was slowly instilled. After the intranasally instilled solution was aspirated, the animals were kept in the upright position for an additional 2 min to allow sufficient spreading of the fluid throughout the airways. Animal welfare was monitored by weighing the animals before each intranasal instillation; no animals needed to be withdrawn from the protocol. Previous studies have demonstrated that guinea pigs challenged with LPS are a good model for COPD, since it induces various inflammatory and pathological changes closely mimicking COPD (22, 32).

#### Guinea pig lung slices.

After the last challenge (24 h), precision-cut lung slices were prepared as described previously (20, 25). The animals were euthanized using an overdose of pentobarbital sodium (Euthasol 20%; Produlab Pharma, Raamsdonkveer, The Netherlands) followed by exsanguination via the aorta abdominalis. The trachea was cannulated, the diaphragm was opened, and the lungs were filled through the cannula at constant pressure with a low-melting-point agarose (Gerbu Biotechnik, Weiblingen, Germany) solution (1.5%) in a buffer containing (in mM) 0.9 CaCl_2_, 0.4 MgSO_4_, 2.7 KCl, 58.2 NaCl, 0.6 NaH_2_PO_4_, 8.4 glucose, 13 NaHCO_3_, 12.6 HEPES, 0.5 sodium pyruvate, 1 glutamine, minimal essential medium (MEM)-amino acids mixture (1:50), and MEM-vitamins mixture (1:100), pH = 7.2. The agarose solution contained 1 μM isoproterenol to prevent postmortem constriction (25). After filling, the lungs were covered with ice for at least 30 min to solidify the agarose for slicing. The lungs were removed, and cylindrical tissue cores were prepared from the lobes using a rotating sharpened metal tube (diameter 15 mm), followed by slicing the tissue in ice-cold buffer composed of 1.8 mM CaCl_2_, 0.8 mM MgSO_4_, 5.4 mM KCl, 116.4 mM NaCl, 1.2 mM NaH_2_PO_4_, 16.7 mM glucose, 26.1 mM NaHCO_3_, 25.2 mM HEPES, and 1 μM isoproterenol, pH = 7.2, using a tissue slicer (Compresstome VF- 300 microtome; Precisionary Instruments, San Jose, CA). Lung slices were cut at a thickness of 500 μm and washed several times to remove the agarose and cell debris from the tissue. Slices were incubated in a 12-well plate overnight in MEM composed of (in mM) 1.8 CaCl_2_, 0.8 MgSO_4_, 5.4 KCl, 116.4 NaCl, 1.2 NaH_2_PO_4_, 16.7 glucose, 26.1 NaHCO_3_, 25.2 HEPES, 0.5 sodium pyruvate, 1 glutamine, MEM-amino acids mixture (1:50), and MEM-vitamins mixture (1:100), pH = 7.2, containing penicillin and streptomycin (1:100), at 37°C in a CO_2_- and humidity-controlled atmosphere.

#### Human lung slices.

Peripheral lung tissue from COPD GOLD 1 (*n* = 4) and GOLD 2 (*n* = 3) patients and from non-COPD control subjects (*n* = 5) was obtained from subjects undergoing surgery for lung cancer using tumor-free tissue far from the tumor site, except one control that was obtained from a nontransplanted donor lung. All tissue was collected according to the Research Code of the University Medical Center Groningen (https://www.umcg.nl/SiteCollectionDocuments/English/Researchcode/UMCG-Researchcode,%20basic%20principles%202013.pdf) and national ethical and professional guidelines (“Code of conduct,” Dutch Federation of Biomedical Scientific Societies, https://www.federa.org). Characteristics of the subjects are shown in Table 1. After placing on a metal plate on ice, 2% low-melting agarose was slowly injected in the tissue, evenly distributed at several sites of the tissue, essentially as described by Sturton et al. (29). Subsequently, the tissue was covered with ice for 15 min. Cylindrical cores of 15 mm in diameter were prepared, cut with a tissue slicer into 500 μm thin slices, and processed as described above for guinea pig lung slices.

**Table 1.** Clinical data

	Control Subjects	COPD Patients
No. of subjects	5*	7
Age, yr^†^	65 (42–69)	66 (42–69)
Men/women^†^	2/2	5/2
Ex/current smoker^†^	3/1	4/3
Pack-years^†^^§^	40 (28–52)	50 (20–54)
FEV_1_, %predicted^†^	109.5 (87–122)	89 (58–115)
FEV_1_/FVC^†^	74.2 (69.4–84.2)	60.1 (52.5–67.3)

Values, except no. of subjects, sex, and smoking status, are medians (ranges). COPD, chronic obstructive pulmonary disease; FEV_1_, forced expiratory volume in 1 s; FVC, forced vital capacity.

* 4 non-COPD patients undergoing surgery for lung cancer and 1 healthy lung donor.

† Data for healthy lung donor not available.

§ Data from 2 control subjects and 2 COPD patients are missing.

#### Airway responsiveness measurements.

After the slices were washed in medium, airway responsiveness to methacholine (10^−9^ to 3 × 10^−3^ M, using cumulative concentrations in half-log increments) was assessed in 1–6 slices/animal or human donor, using video-assisted microscopy (Nikon Eclipse TS 100). To this aim, individual slices were positioned under the microscope using a 24-well plate, mechanically maintained with a Teflon ring of 7 mm inner diameter and 10 mm outer diameter, and covered with 1 ml of MEM. The only slices used were those with approximately circular airways (longest/shortest diameter < 2) and with ciliary beating as an indication of intact epithelium and viability of the slices. In guinea pig slices, methacholine-induced contraction was measured both in large and small airways (in this species defined by diameters larger and smaller than 150 µm, respectively), whereas, in human slices, only small airways (<500 µm) were studied. To quantify airway luminal area, image acquisition software (NIS-Elements; Nikon) was used. Images of the airways were acquired every 2 s during the whole course of the experiment, starting 2 min before the addition of the first dose of contracting agent to allow for baseline measurements of the airway caliber. Airway constriction was then expressed as percentage of the initial (baseline) area of the airway lumen. Per slice, one airway was measured. After the cumulative concentration-response curve was established, slices were thoroughly washed in fresh medium.

#### Histochemistry.

After a subsequent overnight washout, lung slices were placed in cassettes supported by biopsy foam pads, fixed in 10% formalin for 24 h, and embedded in paraffin. Paraffin sections (4 μm thin) were cut from the slices to assess remodeling parameters. Airway smooth muscle mass was determined by α-SMA staining. After deparaffinization, endogenous peroxidase was blocked for 30 min using 0.3% H_2_O_2_ in PBS [(in mM)140 NaCl, 2.6 KCl, 1.4 KH_2_PO_4_, and 8.1 Na_2_HPO_4_, pH7.4). After 5 min washing with PBS, the sections were cooked in 10 mM Na_3_-citrate buffer, pH 6, for 5 min using a pressure cooker. Sections were then incubated for 15 min with 1% Triton X-100 in PBS and washed three times with PBS afterward. The sections were incubated for 2 min with mouse anti-α-smooth muscle actin antibody (Sigma-Aldrich), diluted 1:1,000 in 1% bovine serum albumin (BSA) in PBS. Subsequently, the sections were incubated for 30 min with 1% BSA in PBS and washed three times with PBS. The secondary antibody [horseradish peroxidase-conjugated goat anti-mouse IgG antibody (Sigma-Aldrich), 1:200 in 1% BSA in PBS] was incubated for 30 min and washed three times with PBS. 3,3′-Diaminobenzidine (DAB) was dissolved in PBS at a concentration of 0.34 mg/ml, and 0.3% H_2_O_2_ was added just before use. After 20 min incubation, sections were washed with ultrapure water and counterstained with hematoxylin. After 5 min rinsing under running tap water, sections were dehydrated in ethanol and covered with mounting medium. Airways were digitally photographed and analyzed using Image J software (National Institutes of Health, Bethesda, MD). The positively stained area (μm^2^) was normalized to the square of the basement membrane length (μm^2^). For evaluation of emphysema, paraffin sections were stained with hematoxylin and eosin. Mean linear intercept (MLI) was determined as a measure of alveolar airspace size as described previously (22), using 20–25 photomicroscopic images (magnification, ×200) per slice.

#### Mathematical model.

A multiscale biomechanical model previously developed (12) was used to couple contractile force generated by airway smooth muscle at the cell level and the narrowing of a nonlinearly elastic airway wall embedded in parenchyma. Briefly, this model considers the airway as an axisymmetric thick-walled cylinder of fixed length in a plane-strain approximation (with no axial displacement) and consists of two layers representing the airway wall and the surrounding parenchymal tissue. The parenchyma is assumed to be a compressible linear elastic material with compressibility ν, which can be reduced to mimic connective tissue damage associated with COPD. The airway wall is considered to be an incompressible nonlinear elastic material of modulus represented by the variable γ when parametrized relative to the elastic modulus of the parenchyma at zero stress. As such, an increase in γ may be interpreted as an increase of airway elasticity modulated by matrix structure and/or a degradation of elastin in the parenchyma. The finite thickness χ of the airway wall is normalized by the radius of the lumen. Fibers embedded as rings in the airway wall represent the ASM bundles and the collagen-dominated extracellular matrix; they are thus assumed to passively stiffen as collagen is recruited upon airway inflation (uncrimping of collagen fibers and thus increased load bearing) and to actively generate a contractile force upon ASM activation. The passive stiffness of these fibers is governed by two parameters C_1_ and C_2_: C_1_ takes into account the density of fibers, whereas C_2_ governs the nonlinear increase in the stiffness of the fibers as they stretch. The active contribution of the ASM in the mechanical properties of the airway results from cellular forces generated by contractile units made of a myosin filament and adjacent actin filaments. β is a parameter accounting for the volume fraction of the ASM cells in the airway, the number of parallel myosin filaments within a single ASM cell and also indirectly for the density of receptors within an ASM cell. Multiplying β by the contractile force generated by a single myosin filament determined via the Huxley-Hai-Murphy (HHM) model results in the overall contractile force of the ASM. Note that a fixed prestress is also applied to mimic the inflation of the lung in the preparation of the PCLS. Additionally, displacement and radial stress are assumed to be always continuous at the boundary between the airway wall and the parenchyma.

First, simulations using this model were used to generate concentration-response curves and identify a baseline set of parameters fitting the concentration-response curves from the human and guinea pig control data. Further simulations were then carried out to determine the changes required in the six parameters above to generate similar changes to those observed in the dose-response curves of human COPD- and LPS-challenged guinea pig PCLS. The parameter changes identified in this way enable inference of the structural changes that could have occurred to generate the observations. The MATLAB scripts for running model simulations as described here can be obtained by contacting the corresponding author of Hiorns et al. (12).

#### Data analysis.

Data are expressed as means ± SE. An *n* = 1 represents the average of the measurements of each airway classification per animal. A power analysis (μ1–μ2 = 40, σ = 30, α = 0.5, power = 0.8) determined that nine animals were to be included per group for the constriction experiments. Per group, 10 animals were included to anticipate an experimental dropout rate of 10%, which allowed for housing all guinea pigs in pairs. Statistical differences were determined by two-way ANOVA, by paired or unpaired Student’s *t*-test, or by Mann-Whitney *U*-test as appropriate. Correlations were determined using Pearson’s correlation coefficient test. Differences were considered statistically significant when *P* < 0.05.

## RESULTS

### 

#### Airway responsiveness in guinea pigs.

**Fig. 1.**
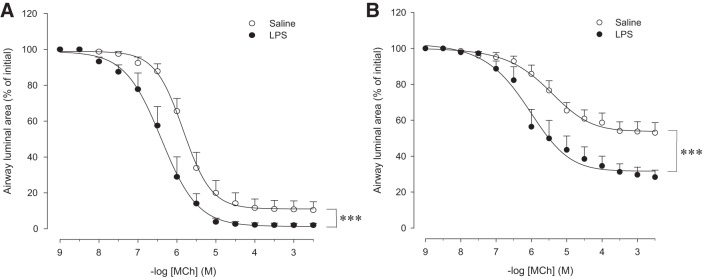
Airway responsiveness toward methacholine (MCh) of large (*A*) and small (*B*) intrapulmonary airways in lung slices obtained from male guinea pigs challenged with either saline or lipopolysaccharide (LPS), two times weekly for 12 wk. Data represent means ± SE of 8–9 animals/group. ***P < 0.001 between curves.

Methacholine induced a concentration-dependent constriction of intrapulmonary large and small airways in PCLS obtained from saline- and LPS-challenged guinea pigs (Fig. 1, *A* and *B*). Interestingly, the maximal constriction (*E*_max_) of the large airways from the saline-challenged controls was 1.8-fold higher compared with that of small airways of the same animals (*P* < 0.001), without a difference in sensitivity (pD_2_) toward methacholine (Table 2). In the large airways of PCLS from LPS-challenged animals, a 4.4-fold higher sensitivity toward methacholine was observed compared with large airways from saline-challenged controls (*P* < 0.01), with a small increase in maximal effect (*P* < 0.05; Fig. 1*A* and Table 2). The maximal constriction of small airways from LPS-challenged animals was 1.5-fold higher compared with saline-challenged controls (*P* < 0.001), without a difference in sensitivity toward methacholine (Fig. 1*B* and Table 2). Airway sizes among saline- and LPS-challenged guinea pigs, respectively, were similar for large (265 ± 16 vs. 264 ± 15 µm) and small (112 ± 5 vs. 111 ± 4 µm).

**Table 2.** Airway responsiveness to methacholine of intrapulmonary large and small airways in lung slices obtained from saline- and LPS-challenged guinea pigs and of intrapulmonary small airways in lung slices obtained from control subjects and COPD patients

	Large Airways		Small Airways	
Group	*E*_max_, %constriction	pD_2_, −log M	*E*_max_, %constriction	pD_2_, −log M
Guinea pig				
Saline challenged	89.7 ± 4.5	5.83 ± 0.12	48.6 ± 5.4^†††^	5.52 ± 0.25
LPS challenged	98.3 ± 1.0*	6.47 ± 0.17**	72.7 ± 3.9***^†††^	5.88 ± 0.26^†^
Human				
Control subjects			45.8 ± 11.1	5.68 ± 0.37
COPD patients			67.9 ± 3.2^#^	5.77 ± 0.18

Values are means ± SE of 8–9 guinea pigs/group, 5 control subjects, and 7 chronic obstructive pulmonary disease (COPD) patients. *E*_max_, maximal effect; pD_2_, −log of the concentration causing 50% effect (−log EC_50_); LPS, lipopolysaccharide.

* *P* < 0.05,

** *P* < 0.01, and

*** *P* < 0.001 compared with saline-challenged guinea pigs.

† *P* < 0.05 and

††† *P* < 0.001 compared with corresponding large airways.

# *P* < 0.05 compared with control subjects.

#### ASM mass and alveolar airspace size in guinea pigs.

**Fig. 2.**
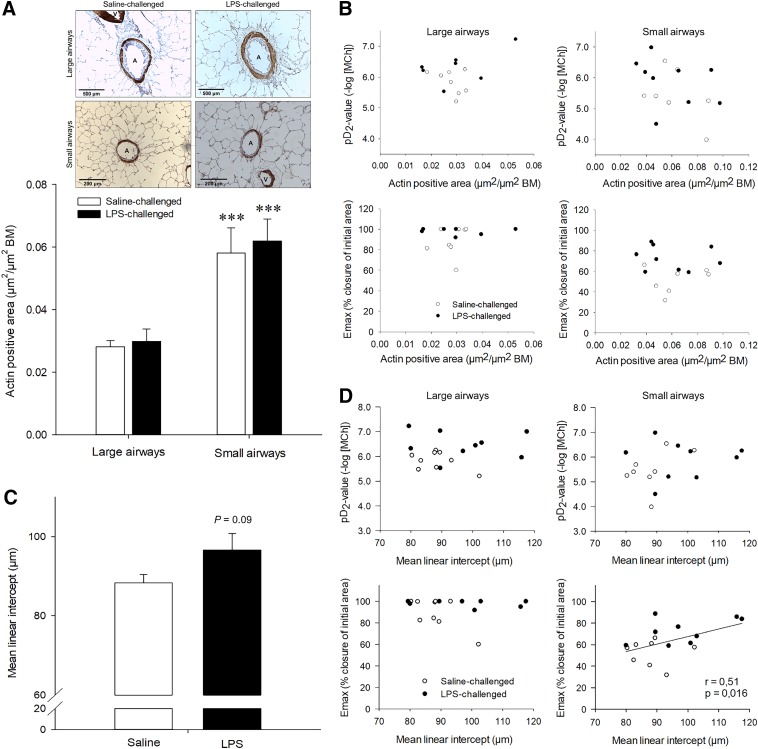
*A*: α-smooth muscle actin (α-SMA)-positive area of large and small airways in lung slices obtained from male guinea pigs challenged with either saline or lipopolysaccharide (LPS), two times weekly for 12 wk. Representative images are shown for each group and airway classification (A, airway; V, vessel). The bar indicates 500 μm for the large airways and 200 μm for the small airways. *B*: correlations between airway smooth muscle mass (α-SMA-positive area) and airway responsiveness [*top*, difference in sensitivity (pD_2_); *bottom*, maximal constriction (*E*_max_)] of large and small airways. BM, basement membrane. Data represent means ± SE of 7–10 animals/group. ***P < 0.001 compared with large airways. *C*: effects of repeated saline or LPS challenge on alveolar airspace size [mean linear intercept (MLI)] in male guinea pig lung slices obtained from guinea pigs challenged with either saline or LPS, two times weekly for 12 wk. *D*: correlations between MLI and airway responsiveness (*top*, pD_2_; *bottom*, *E*_max_) of large and small airways. Data represent means ± SE of 8–10 animals/group.

Compared with saline-challenged controls, repeated LPS challenge did not alter ASM mass of either large or small airways as determined by α-SMA-positive area (Fig. 2*A*). Consequently, no correlations between *E*_max_ or pD_2_ and ASM mass were observed (Fig. 2*B*).

Repeated LPS challenge did induce a trend toward an increase in MLI compared with saline-challenged animals (*P* < 0.10, Fig. 2*C*). Interestingly, a significant correlation between MLI and *E*_max_ of small, but not large, airways was found (Fig. 2*D*). No correlations between MLI and pD_2_ values were observed (Fig. 2*D*).

#### Small airway responsiveness in human PCLS.

**Fig. 3.**
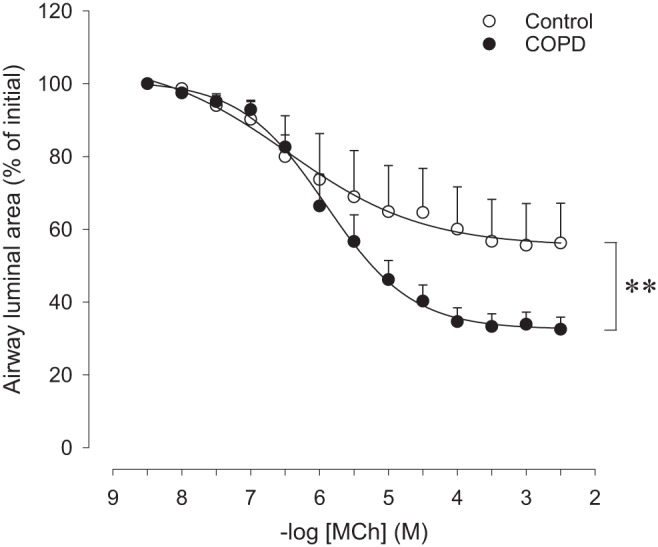
Airway responsiveness toward methacholine (MCh) of peripheral airways in lung slices obtained from control subjects and from patients with chronic obstructive pulmonary disease (COPD). Data represent means ± SE of 5 control subjects and 7 COPD patients. **P < 0.01 between curves.

The responsiveness of human peripheral control airways to methacholine (Fig. 3 and Table 2) was similar to that in the small airways from saline-challenged guinea pigs (Fig. 1*B* and Table 2). A 1.5-fold higher maximal constriction (*P* < 0.05) was observed in lung slices obtained from patients with COPD compared with control subjects, without a difference in pD_2_ (Fig. 3 and Table 2). There was no difference between the airway diameters of control subjects (273 ± 59 μm) and COPD patients (227 ± 48 μm). Remarkably, the hyperresponsiveness of the COPD airways was identical to that observed in the small airways of the LPS-challenged guinea pigs.

#### ASM mass and alveolar airspace size in human PCLS.

**Fig. 4.**
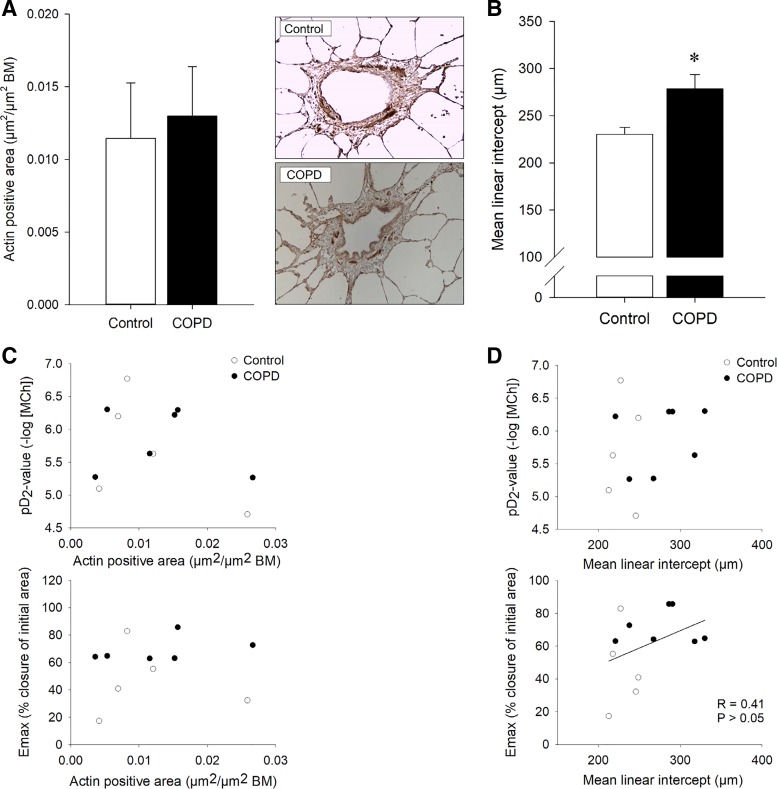
*A*: α-smooth muscle actin (α-SMA)-positive area in lung slices obtained from control subjects and chronic obstructive pulmonary disease (COPD) patients. Representative images are shown for control (*top*) and COPD (*bottom*). Data represent means ± SE of 5 control subjects and 6 COPD patients. *B*: alveolar airspace size [mean linear intercept (MLI)] in lung slices of control subjects and COPD patients. Data represent means ± SE of 5 control subjects and 7 COPD patients. *P < 0.05 compared with control. *C*: correlations between airway smooth muscle mass (α-SMA-positive area) and airway responsiveness (*top*, pD_2_; *bottom*, *E*_max_). *D*: correlations between MLI and airway responsiveness (*top*, pD_2_; *bottom*, *E*_max_).

No difference in ASM mass was found between slices from control subjects and subjects with COPD (Fig. 4*A*). A 1.2-fold higher MLI was measured in the lung slices of the COPD patients compared with the control subjects (*P* < 0.05, Fig. 4*C*). There were no significant correlations between the α-SMA-positive area (Fig. 4*B*) or the MLI (Fig. 4*D*) and the *E*_max_/pD_2_ of methacholine-induced airway constriction.

#### Changes in biomechanical properties in PCLS from COPD patients and LPS-challenged guinea pigs.

**Fig. 5.**
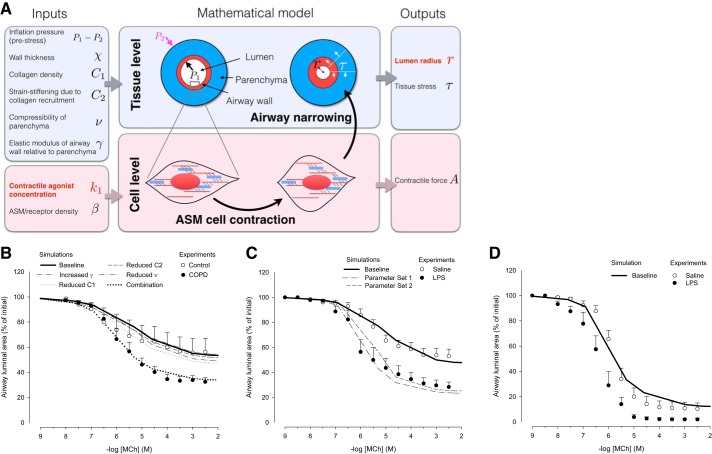
*A*: schematic overview of our previously developed multiscale mathematical model (11) for which a given agonist concentration k1 (model input) causes airway smooth muscle (ASM) cell shortening via actomyosin crossbridge interactions at the cell level, thereby generating airway narrowing at the tissue level. This allows prediction of the lumen radius (*r*) at each concentration (k1) to generate dose-response curves for a given set of parameter values for airway and parenchymal mechanical properties and cell properties listed above. Note that additional outputs are also generated by the model, such as radial and circumferential tissue stresses (τ) and contractile force at the cell level (A), but the most direct comparison with experimental data is via the lumen radius. Multiple simulations were performed in which the parameters were varied within a range until a dose-response curve (k1 vs. *r*) is generated that best fits the baseline/control experimental data. Once this fit (a set of baseline parameters) is obtained, the parameter values are varied one at a time to identify those factors that contribute the most to the modified dose-response curves of the lipopolysaccharide (LPS)/chronic obstructive pulmonary disease (COPD) data. *B*: simulated concentration-contraction curves (solid lines) for human control data and COPD data (from Fig. 3). Inflation pressure for these simulations was set as 0. In the simulation, the effect of an increased γ, a reduced C1, a reduced C2, and a reduced *v* individually and the combination of all the parameters was tested (“combination”). *C*: simulated dose-response curves (solid lines) for saline- and LPS-treated guinea pig slices for small airways with different wall thickness parameters (*parameter sets 1* and *2*; experimental data from Fig. 1*B*). *D*: simulated dose-response curves (solid lines) for saline-treated guinea pig slices for large airways (experimental data from Fig. 1*A*). Parameter values for the simulated curves are given in Table 3.

In the model, the biomechanical properties of the airway wall (ASM and collagen fibers) and parenchyma are characterized by the parameters listed in Fig. 5*A* and Table 3 (12). Any structural changes in the airway or parenchyma would modify these parameters and impact the mechanical response to agonist challenge. Baseline values of these parameters were first obtained by fitting a simulated concentration-contraction curve to the experimental concentration-contraction one from human control subjects (Fig. 5*B* and Table 3). The effect of changing one parameter at a time was then explored. Similar baseline airway diameters and ASM mass measured between control and COPD slices justified keeping the airway wall thickness (χ) and ASM density and/or muscarinic receptor density (β) at baseline values for all human data simulations. Simulating reduction in collagen fiber density within the airway wall (C_1_) or in the extent to which they are recruited (C_2_) generated minimal hyperresponsiveness (Fig. 5*B*). Similarly, reducing the compressibility (ν) or the stiffness (increased γ) of the parenchyma relative to the airway wall generated small levels of hyperresponsiveness (Fig. 5*B*). Mimicking structural changes to the collagen matrix within the airway wall without changes to the parenchyma, and vice versa, thus had a limited impact. In contrast, all of these parametric changes implemented simultaneously induced a significant shift in the simulated dose-response curve, which matched the experimental data obtained on COPD slices (Fig. 5*B* and Table 3). This suggests that structural changes to the matrix occurring in COPD (2) affect both the airway wall and the parenchyma, modifying the effective mechanical properties of both compartments.

**Table 3.** Biomechanical parameters fitted to the experimental dose-response curves using the multiscale biomechanical model of the airway embedded in parenchyma

Airways	Wall Thickness	ASM Density/Muscarinic Receptor Density	Collagen Fiber Density	Strain Stiffening Because of Collagen Recruitment	Compressibility of Parenchyma	Elastic Modulus of Airway Wall Relative to Parenchyma
Human						
** **Control	0.3	5	0.25	1.0	0.4	5
** **COPD	0.3	5	0.05	0.14	0.1	100
Guinea pig						
** **Small airway, saline	0.27	9	0.2	1.0	0.4	2
** **Small airway, LPS (*set 1*)	0.3	9	0.05	0.09	0.1	100
** **Small airway, LPS (*set 2*)	0.38	9	0.05	0.09	0.1	100
** **Large airway, saline	0.4	30	0.03	0.1	0.4	10

Note: parameters fitted to the guinea pig data required higher prestress (inflation pressure). ASM, airway smooth muscle; COPD, chronic obstructive pulmonary disease; LPS, lipopolysaccharide.

For guinea pig slices, model baseline parameters were first fitted to the saline-challenged concentration-response curve for small airways (Fig. 5*C* and Table 3). Higher ASM/muscarinic receptor density (β) and nonzero inflation pressure were required to fit the guinea pig data. Changes in both airway wall and parenchymal properties were needed to match the LPS-challenged guinea pig experimental concentration-response curve for small airways. Specifically, a slightly thicker airway was needed (*parameter set 1* or *2*), together with reduced collagen fiber density and parenchymal compressibility and stiffness relative to the baseline guinea pig parameters (Fig. 5*C* and Table 3). In the case of the saline-treated guinea pig data for large airways, simulated dose-response curves fitted to the experimental dose-response curve (Fig. 5*D* and Table 3) suggested that slightly thicker airways (χ) and significantly increased ASM/muscarinic receptor density (β) are present. The biomechanical model was unable to generate dose-response curves for the LPS-treated guinea pig data for the large airways.

## DISCUSSION

This study successfully shows the high potential of guinea pig and human PCLS in studying (small) airway mechanics in relation to tissue remodeling in COPD. Both in PCLS from a guinea pig model of LPS-induced COPD and in PCLS from patients with mild to moderate COPD, we demonstrated small airway hyperresponsiveness to methacholine, which is not caused by increased ASM mass, but may be related to reduced parenchymal stiffness and compressibility involving increased alveolar airspace size and reduced stiffness of the passive components of the airway wall.

In PCLS from LPS-challenged guinea pigs, a 1.5-fold increase in maximal methacholine-induced airway constriction was observed in the small airways, without a change in sensitivity toward the agonist. Only a small increase in *E*_max_ was observed in the large airways of LPS-challenged animals, due to the pronounced (90%) methacholine-induced constriction of these airways in the saline group. An increase in pD_2_ was observed in large airways. Further studies indicated that emphysema may be involved in LPS-induced small airway hyperresponsiveness, since increased alveolar airspace sizes were positively correlated with the *E*_max_ in small airways within the same PCLS. No correlation between these parameters was found for the large airways; neither was there a correlation between MLI and pD_2_ values for either airway type. From a pharmacological point of view, these results indicate that postreceptor changes are involved in the increased small airway constriction to methacholine, which may at least partially be caused by structural changes in the parenchyma. Because ASM mass was unaltered after LPS challenge for either airway type and there was no correlation between ASM mass and airway responsiveness, ASM mass per se is not an important determinant of AHR in this model.

Remarkably, we found a larger maximal methacholine-induced narrowing of the large airways compared with the small airways, both after saline and LPS challenge. Studies in rat PCLS demonstrated that airway responsiveness (reduction in airway luminal area) toward electrical field stimulation is greater in large airways compared with small airways (28), whereas no differences in the responsiveness to methacholine were observed (19). We did not observe significant differences in sensitivity (pD_2_) toward methacholine between large and small airways in PCLS obtained from saline-challenged guinea pigs. However, in rats, the sensitivity to methacholine is greater in the large airways than in the small airways (19), whereas the opposite has been observed in mice (6). Because different techniques were used in the latter study (6) to determine the constriction of trachea (contractility) and small airways (airway area), the responsiveness (*E*_max_) of large and small airways cannot be compared. The increased responsiveness of the large airways in guinea pig PCLS is not related to the ASM mass, since, similar to human airways (9), the relative ASM thickness was smaller in the large airways. Previous findings using the same animal model demonstrated that repeated LPS challenge did not alter the active tension of isolated airways induced by either methacholine or histamine (T. Pera, R. Gosens, A. Zuidhof, H. Maarsingh, J. Zaagsma, and H. Meurs, unpublished observations), further supporting the contribution of structural changes in the parenchyma to the observed LPS-induced AHR.

This study also describes the first measurements of airway mechanics in PCLS from COPD patients. Remarkably, PCLS of human control subjects demonstrated a similar responsiveness of the peripheral airways to methacholine as the small airways of saline-challenged guinea pigs. This confirms previous observations that guinea pig PCLS are an excellent physiological and pharmacological model for human tissue (25, 27). Similar to the guinea pig data, hyperresponsiveness to methacholine, characterized by an increased *E*_max_, of peripheral airways from patients with mild to moderate COPD (GOLD 1 and 2) was observed. In line with previous studies (13) and the guinea pig model, no changes in ASM mass in the small airways were observed in patients with mild to moderate COPD. Slices from the COPD patients did reveal a small but significantly enhanced MLI; however, no significant correlation between MLI and *E*_max_ was observed. This may be because of both the low number of subjects and the involvement of additional factors, such as microstructural changes in the extracellular matrix components, which may similarly impact the mechanical behavior of the airways and the parenchyma (2).

In the alveolar and small airway walls of COPD patients, reduced expression of elastin and decorin, a proteoglycan involved in the assembly of collagen fibers, and increased expression of collagen have been described (2, 11, 14, 34, 36). Similar observations have been reported for smoke- and LPS-induced guinea pig models of COPD characterized by small airway remodeling and emphysema (4, 15, 22). Because the mechanical properties of collagen fibers highly depend on the quality of their assembly, higher collagen content does not necessarily lead to a stiffer tissue (30). Thus, loss of decorin as observed in COPD (14, 34, 36) will lead to disorganization and sliding of collagen fibers despite an increase of the matrix protein. Indeed, imaging revealed structural differences of parenchymal collagen and elastin between nondiseased subjects and COPD patients (1, 31). Moreover, PCLS studies in mice showed disorganized and stretched parenchymal collagen fibers upon elastin degradation (33). Consequences of extracellular matrix changes for mechanical functions of the lung have been explored in PCLS ex vivo and revealed that airway responsiveness is enhanced upon protease-induced degradation of elastin or collagen fibers (16, 33).

The emerging hypothesis of a central role of extracellular matrix remodeling in AHR (2, 3, 30) is further supported by a biomechanical model (12). The contribution on airway responsiveness of the active ASM cells and the passive airway and parenchymal matrixes could be assessed separately by parametric changes. Simulations of the contraction experiments could fit the COPD experimental data only when changes to passive mechanical properties occurred in both the airway wall and the parenchyma compared with control PCLS. The relative weakening of the extracellular matrix in COPD suggested by these changes, potentially including elastin degradation, decorin loss, and disorganization of collagen fibers, is consistent with both the observation of hyperresponsiveness despite a conserved airway smooth muscle mass and with the increased MLI in COPD patients and LPS-challenged guinea pigs.

Guinea pig has a thicker ASM layer compared with most species, which could explain why the biomechanical model could not generate concentration-contraction curves for the LPS-treated guinea pig data for the large airways and would potentially require distinct baseline characteristics. Indeed, the model parameter fit suggests that, in the control data, both airway wall thickness and ASM density needed to be greater than the corresponding human parameters (Table 3). Another explanation for the failure of the model to simulate the LPS-treated guinea pig data for the large airways is a potentially higher baseline tone that the model is not able to account for. Additionally, any stretch-dependent changes in the parameters (such as decreased tethering in response to strong contraction as a result of LPS-modified extracellular matrix) are not accounted for in the current model but could be an explanation for the strong dose response.

Only few studies reported on small airway structure and responsiveness in PCLS in animal models with characteristics of COPD. Thus, cigarette smoke exposure of rats during 8–16 wk increased the sensitivity but not the *E*_max_ of intrapulmonary airways to carbachol and serotonin, and increased ASM mass, which correlated with the sensitivity to serotonin, but not carbachol (5). Although we did not find an increased ASM mass in our model or in our patients with mild to moderate COPD, the absence of a correlation between ASM mass and hyperresponsiveness to the muscarinic agonist (5) corresponds to our observations. No changes in sensitivity to serotonin or methacholine were found after acute exposure of the rats to cigarette smoke (5), in line with a recent study on acute cigarette smoke exposure in mice (8). In the latter study, a change in the contraction pattern to serotonin, but not to methacholine, was observed that was associated with a change in ryanodine receptor expression (8). Although the acute models were characterized by cigarette smoke-induced inflammation, they are highly unlikely to demonstrate small airway remodeling and emphysema that are important for AHR in COPD. Similarly, airway hyperresponsiveness was not induced in PCLS following acute exposure of mice to LPS, a proinflammatory contaminant from gram-negative bacteria in organic dusts and cigarette smoke that has been associated with the development of COPD and bacterial infection-induced exacerbations of COPD (10, 21, 22). However, chronic LPS exposure induced COPD-like inflammation, small airway remodeling, and emphysema in mice and guinea pigs (22, 32, 35). The present study indicates that biomechanical changes involved in small airway hyperresponsiveness in chronically LPS-exposed guinea pigs translate to small airway hyperresponsiveness in patients with COPD.

Regarding the 3Rs of the use of animals in biomedical research (18), the current method of studying airway responsiveness in PCLS represents both refinement and reduction. Airway responses of small and large airways to (multiple) experimental drugs can be individually measured in different PCLS from the same animal, thus lowering the number of animals needed, whereas refinement is achieved by measuring AHR ex vivo rather than in vivo. Moreover, linking mathematical modeling with the functional studies may lead to future reduction of animals needed to predict outcomes.

In conclusion, this is the first study demonstrating small airway hyperresponsiveness in PCLS from patients with mild to moderate COPD, which appears not to be caused by increased airway smooth muscle mass but rather to be related to reduced parenchymal retraction forces and reduced passive stiffness of the airway wall. In addition, we found evidence that PCLS from chronically LPS-exposed guinea pigs can serve as a useful model to study mechanisms of small airway hyperresponsiveness in mild and moderate COPD.

## GRANTS

This work was supported by Stichting Astma Bestrijding Grant 2010-015 (to H. Maarsingh, R. Gosens, W. Timens, and H. Meurs) and the Medical Research Council Grant MR/M004643/1 UK (to B. S. Brook).

## DISCLOSURES

Part of this study was supported by a grant from Novartis UK.

## AUTHOR CONTRIBUTIONS

H. Maarsingh, C.M.B., B.S.B., R.G., W.T., and H. Meurs conceived and designed research; H. Maarsingh, C.M.B., B.S.B., A.B.Z., C.R.E., M.S., and A.O. performed experiments; H. Maarsingh, C.M.B., B.S.B., A.B.Z., C.R.E., M.S., and A.O. analyzed data; H. Maarsingh, C.M.B., B.S.B., R.G., W.T., and H. Meurs interpreted results of experiments; H. Maarsingh, C.M.B., B.S.B., and A.B.Z. prepared figures; H. Maarsingh, C.M.B., B.S.B., and H. Meurs drafted manuscript; H. Maarsingh, C.M.B., B.S.B., R.G., W.T., and H. Meurs edited and revised manuscript; H. Maarsingh, C.M.B., B.S.B., A.B.Z., C.R.E., M.S., A.O., R.G., W.T., and H. Meurs approved final version of manuscript.
